# Higher frequency of cagA EPIYA-C Phosphorylation Sites in *H. pylori* strains from first-degree relatives of gastric cancer patients

**DOI:** 10.1186/1471-230X-12-107

**Published:** 2012-08-14

**Authors:** Dulciene MM Queiroz, Cícero ISM Silva, Maria HRB Goncalves, Manuel B Braga-Neto, Andréa BC Fialho, André MN Fialho, Gifone A Rocha, Andreia MC Rocha, Sérgio A Batista, Richard L Guerrant, Aldo AM Lima, Lucia LBC Braga

**Affiliations:** 1 Clinical Research Unity – Department of Internal Medicine, University Hospital Walter Cantídio – Federal University of Ceará, P.O. Box: 60430270, Fortaleza, Ceará, Brazil; 2 Laboratory of Research in Bacteriology, Federal University of Minas Gerais, Belo Horizonte, Minas Gerais, Brazil; 3 Center for Global Health, University of Virginia, Charlottesville, VA, USA; 4 Department of Physiology and Pharmacology, Federal University of Ceará, Fortaleza, Ceará, Brazil

**Keywords:** *Helicobacter pylori*, Gastric cancer, *H. pylori* CagA-EPIYA, *H. pylori/vac*A

## Abstract

**Background:**

To evaluate the prevalence of more virulent *H. pylori* genotypes in relatives of gastric cancer patients and in patients without family histories of gastric cancer.

**Methods:**

We evaluated prospectively the prevalence of the infection by more virulent *H. pylori* strains in 60 relatives of gastric cancer patients comparing the results with those obtained from 49 patients without family histories of gastric cancer. *H. pylor*i status was determined by the urease test, histology and presence of *H. pylori ure*A. The cytotoxin associated gene (*cag*A), the *cag*A-EPIYA and vacuolating cytotoxin gene (*vac*A) were typed by PCR and the *cag*A EPIYA typing was confirmed by sequencing.

**Results:**

The gastric cancer relatives were significant and independently more frequently colonized by *H. pylori* strains with higher numbers of CagA-EPIYA-C segments (OR = 4.23, 95%CI = 1.53–11.69) and with the most virulent s1m1 *vac*A genotype (OR = 2.80, 95%CI = 1.04–7.51). Higher numbers of EPIYA-C segments were associated with increased gastric corpus inflammation, foveolar hyperplasia and atrophy. Infection by s1m1 *vac*A genotype was associated with increased antral and corpus gastritis.

**Conclusions:**

We demonstrated that relatives of gastric cancer patients are more frequently colonized by the most virulent *H. pylori cag*A and *vac*A genotypes, which may contribute to increase the risk of gastric cancer.

## Background

*Helicobacter pylori*, a Gram-negative bacterium that infects the stomach of approximately half the world’s population, is associated with the development of gastroduodenal diseases including gastric and duodenal peptic ulcer, distal gastric adenocarcinoma and mucosa-associated lymphoid tissue lymphoma [1]. It is estimated that individuals infected with *H. pylori* have more than two-fold increased risk of developing gastric cancer compared with non-infected ones [2] although Japanese studies might suggest that nearly all gastric cancer is related to *Helicobacter*[3]. Why only 1 to 5% of *H. pylori-*infected persons develop gastric cancer remains unknown and it seems to depend on the relationship between environmental, host genetics and bacterial virulence factors.

Several studies have shown an increased risk of developing gastric cancer in relatives of patients with the disease [2,4]. Similarly, an increased prevalence of precancerous gastric lesions has been observed in relatives of gastric cancer patients [5]. However, molecular mechanisms by which *H. pylori* triggers the process leading to gastric carcinoma remain largely unknown.

The most investigated *H. pylor*i virulence determinant, the *cag-*PAI (cytotoxin associated gene pathogenicity island), encodes a type IV secretion system (T4SS) that is responsible for the entrance of an effector protein, CagA, into host gastric epithelial cells [6,7]. Once translocated, CagA localizes to the inner surface of the plasma membrane where it is phosphorylated on the tryrosine residues within phosphorylation motifs in carboxy-terminal variable region of the protein by multiple members of the src-family tyrosine kinases. Once phosphorylated, CagA forms a physical complex with SHP-2 phosphatase and triggers abnormal cellular signals, which enhance the risk of damaged cells acquiring precancerous genetic changes [8,9].

The phosphorylation motifs, defined as a sequence of five amino acids (Glu-Pro-Ile-Tyr-Ala), are classified as EPIYA-A, EPIYA-B, EPIYA-C and EPIYA-D, according to amino acid sequences flanking the motifs. CagA proteins nearly always possess EPIYA-A and -B segments, that are followed by none, one, two or three C segments in strains circulating in the Western countries, or a D segment, in East Asia strains [10,11]. It has also been shown that infection with CagA strains having high number of EPIYA-C segments imparts a greater risk of precancerous gastric lesions and cancer [12-15].

Another virulence factor of *H. pylori* is a protein known as vacuolating cytotoxin A (VacA), which causes cytoplasmatic vacuolization in gastric epithelial cells, increasing the plasma cell and mitochondrial membrane permeability leading to apoptosis. The production of the cytotoxin is associated with the cag-PAI but depends on the *vac*A genotype [16-18]. The *vac*A is a polymorphic gene with two main signal region genotypes s1 and s2, and two different alleles in the mid region of the gene named m1 and m2. Infection with strains possessing the s1m1 genotype has been associated with precancerous gastric hypochlorhydria [17] and gastric carcinoma [19].

In a recent study conducted in Fortaleza, Northeastern, Brazil, in an area of high prevalence of gastric cancer and *H. pylori* infection, our group has shown a high prevalence of either pangastritis or precancerous lesions in relatives of gastric cancer patients infected with *H. pylori*[20].

Furthermore, Argent *et al*., (2008) observed an association between *vac*A s1m1 genotype of *H. pylori* strains and low gastric acid secretion in first-degree relatives of gastric cancer patients from Scotland [21]. Otherwise, the authors did not find associations between CagA positive status and or number of tyrosine phosphorylated motifs and gastric lesions in that population.

Since geographical differences have been observed among studies that evaluated association between *H. pylori* virulence factors and diseases, the aim of this cross-sectional prospective study was to evaluate the CagA EPIYA motifs of *H. pylori* strains in first-degree relatives of gastric cancer patients comparing the results with those obtained from a control group composed of subjects with no family history of gastric cancer. Because the s1m1 genotype of the *vac*A *H. pylori* was seen to be more frequently observed in the strains of gastric cancer patients, we also evaluated the *vac*A mosaicism in the strains.

## Methods

The study was approved by the Ethical Committee of Research of the University of Ceará, and informed consent was obtained from each subject.

### Patients

Sixty *H. pylori*-positive first-degree relatives [42 female; mean age 40.42 ± 11.80; (4 brothers and 13 sisters; mean age 56.24 ± 11.80 years, 14 sons and 29 daughters; mean age 34.51 ± 7.66)] of gastric cancer patients from outpatient follow-up at Walter Cantídio Hospital were invited to participate. The control group was composed of 49 (32 female; mean age 43.20 ± 12.59) *H. pylori*-positive patients who concurrently underwent upper gastrointestinal endoscopy for investigation of dyspepsia at the same Hospital. They did not have family history of gastric cancer, and were social class matched with the study group. Patients with history of gastric surgery, active gastrointestinal bleeding, use of steroids, immunosuppressive drugs, NSAIDs, proton pump inhibitors or who were treated for *H. pylori* eradication were excluded from the study. Relatives and controls were not included if they were under 18 or above 81 years old.

### Biopsy fragment collection

Gastric fragments were obtained during endoscopy from five different sites as recommended by the Updated Sydney System for classification of gastritis [22]. Additionally, two fragments were collected from the antral mucosa for the rapid urease test and for DNA to investigate the presence of *H. pylori* genes. *H. pylori* infection was confirmed by positive results in at least two tests including a rapid urease test, histological analysis and presence of *ure*A gene of *H. pylori*.

### Histology

Endoscopic biopsy samples of the gastric mucosa were fixed in 10% formalin and embedded in paraffin wax, and 4-μm-sections were stained with hematoxylin-eosin for routine histology. Gastritis was classified according to the Updated Sydney system. The samples of the gastric mucosa were also stained with Giemsa for detection of *H. pylori.*

### DNA extraction

The antral gastric DNA was extracted using the QIAmp (QIAGEN, Hilden, Germany) kit according to the manufacturer’s recommendations with minor modifications [23]. The DNA concentration was determined by spectrophotometry using NanoDrop 2000 (Thermo Scientific, Wilmington, NC) and stored at −20°C until use.

The presence of *H. pylori* specific *ure*A gene was evaluated according to methodology reported by Clayton *et al.*, [24]*.* The standard Tx30a *H. pylori* strain was used as a positive control, and an *Escherichia coli* strain and distilled water were both used as negative controls.

The thermocycler GeneAmp PCR System 9700 (Applied Biosystems, Foster City, CA) was used for all reactions. The amplified products were electrophoresed in 2% agarose gel, stained with ethidium bromide, and analyzed in an ultraviolet light transilluminator.

### *vac*A and *cag*A detection

PCR amplification of the *vac*A signal sequence and mid region was performed by using the oligonucleotide primers described by Atherton *et al*., [15]. The strains were initially classified as type s1 or s2 and type m1 or m2. All *H. pylori* strains with s1 were further characterized into s1a, s1b or s1c [25,26]*.*

The *cag*A gene was amplified by means of two previously described set of primer pairs [27,28]. A *H. pylori* strain from our collection (1010–95), known to be *vac*A s1m1 and *cag*A-positive, was used as a positive control, and the s2m2 *vac*A genotype, *cag*A-negative standard Tx30a *H. pylori* strain and distilled water were both used as negative controls. The *H. pylori* strains were considered to be *cag*A-positive when at least one of the two reactions was positive.

### Amplification of the 3’ variable region of *cag*A

**Figure 1:**
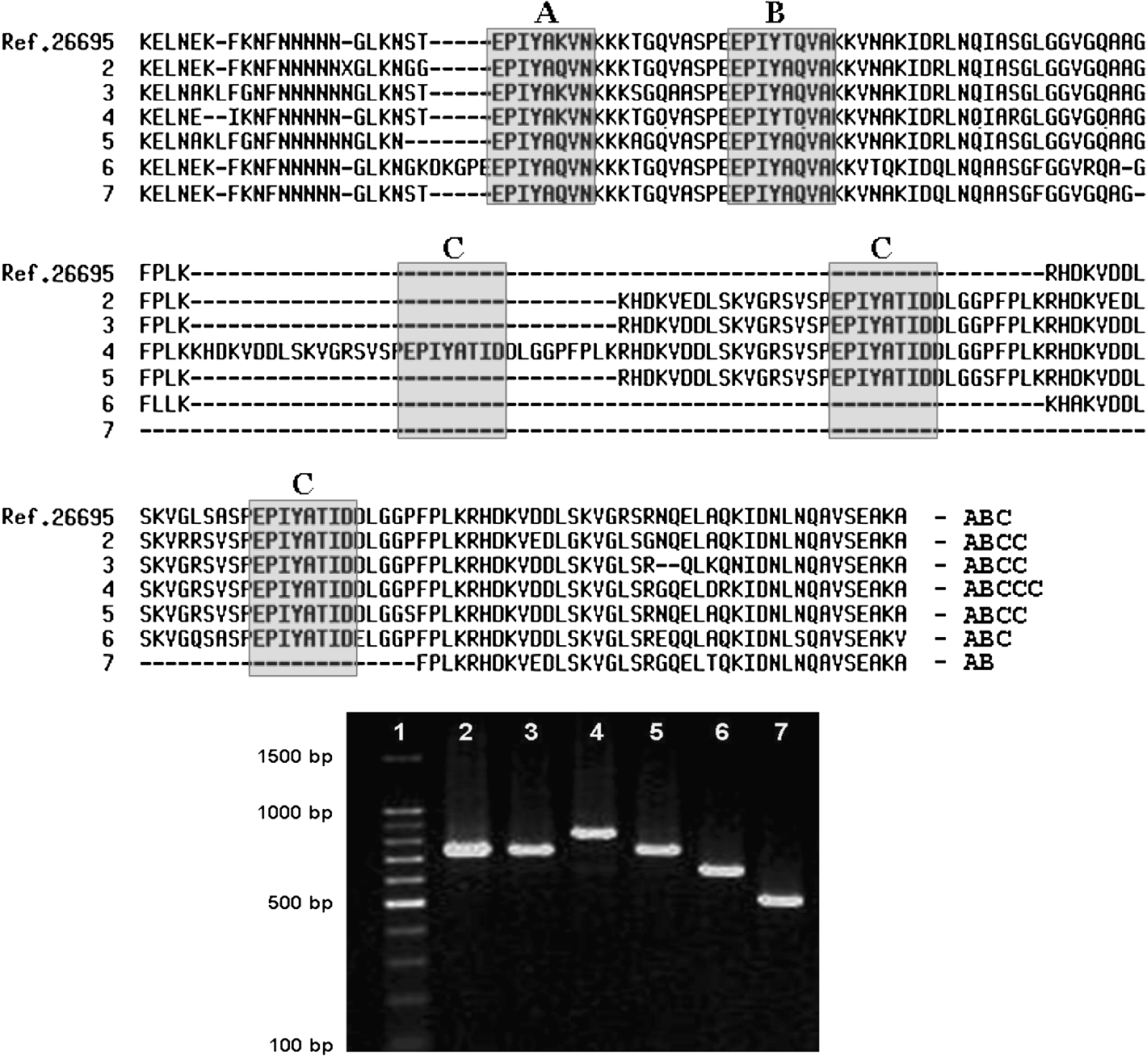
**Electrophoresis of representative samples with different CagA EPIYA patterns seen in relatives of gastric cancer patients and controls.** Columns 2, 3 e 5: EPIYA-ABCC (740 bp); column 4: EPIYA-ABCCC (850 bp); column 6: EPIYA-ABC (640 bp) and Column 7: EPIYA-AB (500 bp). Upper: partial alignment of amino acid sequencing of the carboxy-terminal CagA strains and a reference strain (*H. pylori* 26695).

For the PCR amplification of the 3’ variable region of the *cag*A gene (that contains the EPIYA sequences), 20 to 100 ng of DNA were added to 1% Taq DNA polymerase buffer solution (KCl 50 mM and Tris–HCl 10 mM, pH, 8.0), 1.5 mM MgCl_2_, 100 μM of each deoxynucleotide, 1.0 U Platinum Taq DNA polymerase (Invitrogen, São Paulo, Brazil), and 10 pmol of each primer, for a total solution volume of 20 μL. The primers used were previously described by Yamaoka *et al*. [29]. The reaction conditions were: 95°C for 5 minutes, followed by 35 cycles of 95°C for 1 minute, 50°C for 1 minute, and 72°C for 1 minute, ending with 72°C for 7 minutes. The reaction yielded products of 500 to 850 bp as follows: EPIYA-AB: 500 bp; EPIYA-ABC: 640 bp; EPIYA-ABCC: 740 bp and EPIYA-ABCCC: 850 bp (Figure 1).

We also used the method described by Argent *et al*. [30] for the PCR amplification of the 3’ variable region of the *cag*A gene that contains the EPIYA sequences in order to improve the accuracy of our results.

### Sequencing of the 3’ variable region of *cag*A

A subset of samples was randomly selected for sequencing in order to confirm the PCR results. PCR products were purified with the Wizard SV Gel and PCR Clean-up System (Promega, Madison, MI) according to the manufacturer’s recommendations. Purified products were sequenced using a BigDye Terminator v3.1 Cycle Sequencing kit in an ABI 3130 Genetic Analyzer (Applied Biosystems, Foster City, CA). The sequences obtained were aligned using the CAP3 Sequence Assembly Program (available from: http://pbil.univ-lyon1.fr/cap3.php). After alignment, nucleotide sequences were transformed into amino acid sequences using the Blastx program (available from: http://blast.ncbi.nlm.nih.gov/Blast.cgi) and compared to sequences deposited into the GenBank (http://www.ncbi.nlm.nih.gov/Genbank/).

### Statistical analysis

Data were analyzed with SPSS (Inc. Chicago, IL), version 17.0. The risk of relatives of gastric carcinoma to be infected by more virulent strains, with increased number of EPIYA-C motifs and s1m1 *vac*A genotype, was initially evaluated in univariate analysis. For that, *cag*A strains were stratified in those possessing at least one EPIYA–C segment and those with more than one EPIYA-C segment and the most virulent *vac*A s1m1 genotype was compared with s1m2 plus s2m2. Variables with a *p*-value less-than or equal to 0.25 were included in the final model of logistic regression, controlling for the influences of age and sex. Odds Ratio (OR) and 95% confidence intervals (CI) were calculated. The logistic model fitness was evaluated with the Hosmer-Lemeshow test [31]. Association of the number of EPIYA-C segments and the presence of *vac*A virulent genotypes with the degree of gastric inflammation, atrophy and intestinal metaplasia was done by the two-tailed Mann–Whitney Test. The level of significance was set at a *p* value ≤0.05.

## Results

The presence of *H. pylori* specific *ure*A gene was detected in the gastric mucosa of all 109 studied subjects.

### *cag*A status of the patients

*cag*A positivity was observed in the gastric fragments from 51 (85.00%) of 60 gastric cancer relatives and in those from 43 (87.76%) of 49 controls, without difference between the groups (*p* = 0.68; OR = 1.26, 95%CI = 0.37 – 4.40).

### The number of EPIYA-C segments

The EPIYA pattern of all *cag*A-positive strains from both relatives of gastric cancer patients and controls were successfully typed. The Yamaoka methodology allowed the detection of mixed strain infection. The concordance between the methods used was almost 100%. The results were confirmed by sequencing of the 3’ variable region of *cag*A in 30 randomly selected PCR products.

Four patterns of EPIYA motifs were found: AB, ABC, ABCC, and ABCCC. No Asian EPIYA-D motif was observed. The distribution of the EPIYA genotypes is shown in the Table 1.

**Table 1:** **Distribution of EPIYA genotypes in the gastric cancer relatives (n = 51) and controls (n = 43) colonized by a*cag*A-positive strains**

**EPIYA Genotype**	**Control group n (%)**	**Gastric cancer relatives**	
		**Siblings n (%)**	**offspring n (%)**
EPIYA-AB	03 (7.0)	0	0
EPIYA-ABC	32 (74.4)	09 (60.0)	20 (55.5)
EPIYA-ABCC	07 (16.3)	04 (26.7)	12 (33.2)
EPIYA-ABCCC	01 (2.3)	01 (6.7)	02 (5.6)
EPIYA-ABC + ABCC	0	01 (6.7)	02 (5.6)
Total	43 (100.0)	15 (100.0)	36 (100.0)

### *vac*A mosaicism distribution

The distribution of *vac*A genotypes is shown in the Table 2. In 59 cases (54.13%) the *vac*A genotype was s1m1, in 35 (32.11%) it was s1m2 and 6 (5.50%) s2m2. In three (2.75%) cases two *vac*A genotypes were observed and in six (5.50%) only the signal sequence (s1) was detected. DNA was not enough to genotype m allele in four among these cases and in two, m was not typable. In all cases with s1 strains they were genotyped as s1b, except in one case who was colonized by s1a and s1b strains.

**Table 2:** **Distribution of*vac*A alleles of*H. pylori*strains of relatives of gastric cancer patients (n = 55) and control group (n = 48)**

***vac*A Genotypes**^1^	**Control group n (%)**	**Gastric cancer relatives**	
		**Siblings n (%)**	**Offspring n (%)**
s1m1	23 (47.92)	10 (66.67)	26 (65.00)
s1m2	21 (43.75)	04 (26.67)	10 (25.00)
s2m2	03 (6.25)	01 (6.67)	02 (5.00)
Mixed^2^	01 (2.08)	0	02 (5.00)
Total	48 (100)	15 (100)	40 (100)

^1^Only *vac*A s1 genotype was identified in 6 cases (1 control, 2 siblings and 3 offsprings of gastric cancer relatives); ^2^Mixed infection by s1m1 and s2m2 or s1m1 and s1m2 (two cases).

Infection by the most toxigenic *vac*A genotype (s1m1) was more frequently observed in the gastric cancer relatives (65.45%) than in the controls (47.92%). When s and m alleles were individually evaluated, no difference in the frequency of s1 allele was observed between the groups, but m1 allele was more frequently observed in the gastric cancer relatives.

### Association among the number of EPIYA-C motifs and the *vac*A s1m1 genotype and family history of gastric cancer

The relatives of gastric cancer patients were significantly and independently more frequently colonized by *H. pylori* strains with increased number CagA-EPIYA-C segments and with the most virulent s1m1 *vac*A genotype even after adjustment for age and gender (Table 3).

**Table 3:** Covariables associated with gastric cancer in the first-degree relatives of gastric cancer patients in comparison with subjects without family history of gastric cancer

**Variables**	**Univariate analysis**	**Multivariate analysis**		
	***p***	**OR**	**95% CI**	***p***
Gender	0.27	–	–	–
Age	0.30	–	–	–
> 1 EPIYA-C motif	0.01	4.23	1.53 – 11.69	0.006
s1m1 *vac*A allele	0.17	2.80	1.04 – 7.51	0.04

The Hosmer-Lemeshow test was fit (8 degrees of freedom, p > 0.20, with 10 steps).

No difference was observed between siblings and offspring in respect to infection by strains containing an increased number of EPIYA-C motifs (*p* = 0.98; OR = 1.20, 95%CI = 0.30 – 4.86) and the *vac*A genotypes s1m1 vs. s1m2 and s2m2 (*p* = 0.84; OR = 0.92, 95%CI = 0.22 – 3.97) as shown in the Tables 1 and 2.

### Associations among the number of EPIYA-C segments and *vac*A genotypes and gastric histological alterations

The degrees of corpus gastritis (*p* = 0.04), antrum activity (*p* = 0.01) and corpus activity were significantly higher in the relative of gastric cancer patients than in the control group.

A higher number of EPIYA-C segments was associated with gastric corpus inflammation (*p* = 0.04), gastric corpus foveolar hyperplasia (*p* = 0.05) and gastric corpus atrophy (*p* = 0.05) in the relatives of gastric cancer patients.

Infection by the most virulent *vac*A s1m1 genotype was associated with more marked antral (*p* = 0.03) and corpus (*p* = 0.05) gastritis, when both groups were evaluated together.

## Discussion

*H. pylori* infection is recognized as the most important risk factor for distal gastric cancer. Furthermore, the increased rates of the disease in relatives of gastric cancer points to host genetics and/or share of the most *H. pylori* virulence strains as risk factors.

In this study, we demonstrated that relatives of gastric cancer patients are more frequently colonized by *H. pylori* strains with the most virulent *vac*A genotype, s1m1, and by CagA-positive strains possessing a higher number of EPIYA-C segments than the *H. pylori* strains of the patients without a family history of the disease.

Although no previous study has demonstrated that gastric cancer relatives are more frequently colonized by more virulent *H. pylori* strains, infection by *vac*A s1m1 was associated with low gastric acid secretion, a precancerous condition, in first-degree relatives of Scottish gastric cancer patients [21]. Otherwise, no association between the gastric acid secretion and the number of CagA EPIYA-C segments was observed by the authors [21].

CagA is the first bacterial oncoprotein to be identified [32]. The protein is delivered into the gastric epithelial cell through a bacterial T4SS and localizes to the inside of the cell membrane, where it is phosphorylated by host cell kinases. Upon phosphorylation, the EPIYA-C segment interacts with SHP-2 phosphatase, a bona fide oncoprotein that is associated with a series of human cancers. The higher the number of EPIYA-C segments, the higher the affinity for SHP-2 which is required for a full activation of ERK/MAPK pathway.

Infection with CagA strains possessing higher number of EPIYA-C segments has been associated with precancerous gastric lesions and gastric cancer in Caucasian [11-13,30] and Brazilian populations [15].

It is well established that *H. pylori* infection is predominantly acquired in childhood and that the infection often persists for life unless treated. Epidemiological data and genetic analysis of *H. pylori* strains have demonstrated that the strains are usually acquired within the family. In fact, infected mother and infected siblings are the main risk factors for the acquisition of the infection [33,34] and genetic fingerprint methods have demonstrated genetic homogeneity in the *H. pylori* strains within the families. Based on these findings and the results of the present study, we may hypothesize that first degree relatives of gastric cancer patients may share more virulent *H. pylori* strains that may increase the risk of gastric cancer.

As noted above, first-degree relatives of gastric cancer patients also share the same or similar genetic background that may increase the risk of gastric cancer. Polymorphisms in genes coding pro-inflammatory cytokines, such as interleukin 1 beta (IL-1β), interleukin-1 receptor antagonist (IL1Ra) and tumor necrosis factor-alpha (TNF-α) are accepted as risk factors of gastric cancer, depending on the geographic region [35-39]. It has also been demonstrated that having increasing number of pro-inflammatory genotypes [36,37], as well as a concomitant infection by more virulent *H. pylori* strains progressively increases the risk of gastric precancerous lesions and cancer [39].

## Conclusions

In conclusion, we demonstrated that relatives of gastric cancer patients are more frequently colonized by the most virulent *H. pylori cag*A and *vac*A genotypes, which may, in addition to human genetic predispositions, further increase their risk of gastric cancer, thus providing additional reasons to better understand these infections and perhaps their targeted eradicative treatment.

## Competing interests

The authors declare no-confllict-of-interest.

## Authors’ contributions

DMMQ supervised laboratory work and analyzed the data critical writing and reviewing manuscript., SAB performed DNA extraction, PCR and sequencing and statistical analysis, GAR and AMCR, participated in implementation of the study and wrote the manuscript, CISMS and MHB performed DNA extraction, PCR and database management, MBN, ABF and AMN participated in implementation of the study, data collection, database management and statistical analysis. RLG and AAML performed critical analysing of the data and reviewing of the manuscript. LLBCB participated in conception, design, implementation, coordination of the study and contributed to manuscript writing critical writing and reviewing. All authors have read and approved the final version of the manuscript.

## Pre-publication history

The pre-publication history for this paper can be accessed here:

http://www.biomedcentral.com/1471-230X/12/107/prepub
